# Toward genome assemblies for all marine vertebrates: current landscape and challenges

**DOI:** 10.1093/gigascience/giad119

**Published:** 2024-01-27

**Authors:** Emma de Jong, Lara Parata, Philipp E Bayer, Shannon Corrigan, Richard J Edwards

**Affiliations:** Minderoo OceanOmics Centre at UWA, Oceans Institute, University of Western Australia, Perth, 6009, Australia; Minderoo OceanOmics Centre at UWA, Oceans Institute, University of Western Australia, Perth, 6009, Australia; Minderoo OceanOmics Centre at UWA, Oceans Institute, University of Western Australia, Perth, 6009, Australia; Minderoo Foundation, Perth, 6009, Australia; Minderoo OceanOmics Centre at UWA, Oceans Institute, University of Western Australia, Perth, 6009, Australia; Minderoo Foundation, Perth, 6009, Australia; Minderoo OceanOmics Centre at UWA, Oceans Institute, University of Western Australia, Perth, 6009, Australia; Evolution and Ecology Research Centre, School of Biotechnology and Biomolecular Sciences, University of New South Wales, Sydney, 2052, Australia

**Keywords:** genomics, vertebrates, marine, reference genome, Actinopterygii, Chondrichthyes, biodiversity

## Abstract

Marine vertebrate biodiversity is fundamental to ocean ecosystem health but is threatened by climate change, overharvesting, and habitat degradation. High-quality reference genomes are valuable foundational scientific resources that can inform conservation efforts. Consequently, global consortia are striving to produce reference genomes for representatives of all life. Here, we summarize the current landscape of available marine vertebrate reference genomes, including their phylogenetic diversity and geographic hotspots of production. We discuss key logistical and technical challenges that remain to be overcome if we are to realize the vision of a comprehensive reference genome library of all marine vertebrates.

## Background

Reference genomes have become a fundamental tool for modern biology: reference genome-enabled applications have driven discoveries across medicine and health care, agriculture, biodiversity, ecology, conservation, and evolution. Reference genomes have been economically, logistically, technically, and computationally challenging to produce, leading to reliance on select model organisms to inform genomics-based research. In recent years, advances in sequencing technology and computational tools have facilitated the rapid and affordable production of reference genomes for nonmodel organisms across the tree of life, with ambitious global efforts under way to compile reference genomes for all eukaryotes [1]. The enhanced capacity for large-scale production of reference genomes is timely, as inferences from reference genome-enabled research can inform conservation management practice in this period of unprecedented biodiversity loss and ecosystem decline [2]. Here, we discuss the current landscape of available reference genomes with a focus on marine vertebrates in light of global recognition of the critical role that the oceans and marine biodiversity play in stabilizing our climate and supporting a blue economy [3].

## Reference Genomes Are Unavailable for Over 96% of Marine Vertebrate Species

At the time of writing this article, we assessed the number and phylogenetic diversity of reference genomes currently available for marine vertebrate species. Metadata for assemblies categorized as reference level were obtained from the National Center for Biotechnology Information (NCBI) via their Datasets command line tool using “chordates” as the query (accessed 01/08/2023). Resulting entries were cross-referenced with all known marine vertebrate species (*n* = 19,800) from the World Register of Marine Species [4], yielding a final dataset of 697 assemblies representing 688 unique species (Supplementary Table S1). Eighty-four percent of marine vertebrate orders are represented by at least 1 species (78/93, Table 1), highlighting the progress of existing global consortia with early strategies to target order-level representatives [5]. Representation rapidly diminishes at lower taxonomic levels, however, covering only 41% of marine vertebrate families, 12% of genera, and 3.5% of species. Perciformes, the most speciose vertebrate order, has the highest number of reference genomes, yet still only 39% of Perciformes families are represented. Furthermore, orders with the highest percentage of threatened species according to the IUCN Red List are among the least represented. For example, 47 Rhinopristiformes species are listed as threatened [6], yet currently only 2 species from this order are represented by a reference genome. These data emphasize the need for continued efforts to capture the rich diversity of marine vertebrates, particularly the most vulnerable taxa that are likely to be of high conservation value.

**Table 1:** Summary of the NCBI-listed reference genomes available for marine vertebrates by Order

Class	Order	Marine species	Species with reference genome	Percentage with reference genome	IUCN Red List % threatened^1^ [6]
*Myxini*	Myxiniformes	89	1	1.1	11.8
*Petromyzonti*	Petromyzontiformes	9	4	44.4	21.1
*Elasmobranchii*	Carcharhiniformes	300	6	2	32.9
*Elasmobranchii*	Echinorhiniformes	2	0	0	NA
*Elasmobranchii*	Heterodontiformes	9	0	0	0.0
*Elasmobranchii*	Hexanchiformes	6	0	0	14.3
*Elasmobranchii*	Lamniformes	16	2	12.5	66.7
*Elasmobranchii*	Myliobatiformes	213	2	0.9	48.6
*Elasmobranchii*	Orectolobiformes	46	6	13	37.8
*Elasmobranchii*	Pristiophoriformes	10	0	0	0.0
*Elasmobranchii*	Rajiformes	317	2	0.6	15.5
*Elasmobranchii*	Rhinopristiformes	87	2	2.3	72.3
*Elasmobranchii*	Squaliformes	145	2	1.4	22.1
*Elasmobranchii*	Squatiniformes	26	0	0	59.1
*Elasmobranchii*	Torpediniformes	70	0	0	42.9
*Holocephali*	Chimaeriformes	58	2	3.4	7.6
*Actinopteri*	Acanthuriformes	444	22	5	NA
*Actinopteri*	Acipenseriformes	16	2	12.5	92.6
*Actinopteri*	Acropomatiformes	284	1	0.4	NA
*Actinopteri*	Albuliformes	11	2	18.2	10.0
*Actinopteri*	Alepocephaliformes	142	0	0	NA
*Actinopteri*	Anabantiformes	2	0	0	NA
*Actinopteri*	Anguilliformes	1,010	10	1	0.9
*Actinopteri*	Argentiniformes	97	1	1	NA
*Actinopteri*	Ateleopodiformes	14	1	7.1	0.0
*Actinopteri*	Atheriniformes	110	5	4.5	43.6
*Actinopteri*	Aulopiformes	296	1	0.3	0.0
*Actinopteri*	Batrachoidiformes	78	2	2.6	18.4
*Actinopteri*	Beloniformes	184	3	1.6	13.1
*Actinopteri*	Beryciformes	125	4	3.2	1.4
*Actinopteri*	Blenniiformes	951	2	0.2	NA
*Actinopteri*	Callionymiformes	214	2	0.9	NA
*Actinopteri*	Carangaria incertae sedis	85	3	3.5	NA
*Actinopteri*	Carangiformes	176	14	8	NA
*Actinopteri*	Centrarchiformes	171	2	1.2	NA
*Actinopteri*	Cichliformes	5	1	20	NA
*Actinopteri*	Clupeiformes	317	11	3.5	6.9
*Actinopteri*	Cypriniformes	5	0	0	24.3
*Actinopteri*	Cyprinodontiformes	24	3	12.5	40.3
*Actinopteri*	Dactylopteriformes	14	1	7.1	NA
*Actinopteri*	Elopiformes	9	2	22.2	11.1
*Actinopteri*	Eupercaria incertae sedis	1,784	28	1.6	NA
*Actinopteri*	Gadiformes	645	45	7	2.6
*Actinopteri*	Galaxiiformes	8	0	0	NA
*Actinopteri*	Gobiesociformes	177	2	1.1	11.7
*Actinopteri*	Gobiiformes	1,614	13	0.8	11.0
*Actinopteri*	Gonorynchiformes	6	1	16.7	14.7
*Actinopteri*	Holocentriformes	93	4	4.3	NA
*Actinopteri*	Kurtiformes	372	11	3	NA
*Actinopteri*	Lampriformes	27	4	14.8	0.0
*Actinopteri*	Lophiiformes	406	3	0.7	2.0
*Actinopteri*	Mugiliformes	72	5	6.9	1.9
*Actinopteri*	Mulliformes	100	1	1	NA
*Actinopteri*	Myctophiformes	267	2	0.7	0.0
*Actinopteri*	Notacanthiformes	28	1	3.6	0.0
*Actinopteri*	Ophidiiformes	562	3	0.5	2.2
*Actinopteri*	Osmeriformes	33	6	18.2	30.4
*Actinopteri*	Ovalentaria incertae sedis	798	10	1.3	NA
*Actinopteri*	Perciformes	3,243	168	5.2	9.8
*Actinopteri*	Pleuronectiformes	792	19	2.4	1.8
*Actinopteri*	Polymixiiformes	11	1	9.1	0.0
*Actinopteri*	Saccopharyngiformes	28	0	0	0.0
*Actinopteri*	Salmoniformes	52	10	19.2	47.4
*Actinopteri*	Scombriformes	266	9	3.4	NA
*Actinopteri*	Scorpaeniformes	13	0	0	3.2
*Actinopteri*	Siluriformes	121	37	30.6	14.4
*Actinopteri*	Stomiiformes	451	1	0.2	0.0
*Actinopteri*	Stylephoriformes	1	1	100	NA
*Actinopteri*	Syngnathiformes	311	26	8.4	6.0
*Actinopteri*	Tetraodontiformes	415	9	2.2	4.3
*Actinopteri*	Trachichthyiformes	68	6	8.8	NA
*Actinopteri*	Zeiformes	34	2	5.9	0.0
*Coelacanthi*	Coelacanthiformes	2	1	50	100.0
*Aves*	Accipitriformes	1	1	100	22.6
*Aves*	Anseriformes	48	7	14.6	14.7
*Aves*	Charadriiformes	273	30	11	13.2
*Aves*	Ciconiiformes	12	1	8.3	25.0
*Aves*	Coraciiformes	3	1	33.3	9.1
*Aves*	Falconiformes	5	3	60	12.1
*Aves*	Gaviiformes	5	1	20	0.0
*Aves*	Gruiformes	1	0	0	25.4
*Aves*	Pelecaniformes	61	10	16.4	16.4
*Aves*	Podicipediformes	15	2	13.3	21.7
*Aves*	Procellariiformes	138	10	7.2	44.9
*Aves*	Sphenisciformes	20	18	90	50.0
*Crocodylia*	NA	2	1	50	NA
*NA*	Sauria	1	0	0	NA
*NA*	Squamata	84	6	7.1	16.7
*NA*	Testudines	7	4	57.1	63.0
*Mammalia*	Carnivora	44	14	31.8	26.3
*Mammalia*	Cetartiodactyla	90	33	36.7	36.3
*Mammalia*	Sirenia	4	2	50	80.0

I UCN threatened species include critically endangered, endangered, and vulnerable species. Red text highlights those orders where % threatened species > % with an available reference genome.

## Available Genomes Are Predominantly Derived from Short-Read Sequencing Technology

**Figure 1:**
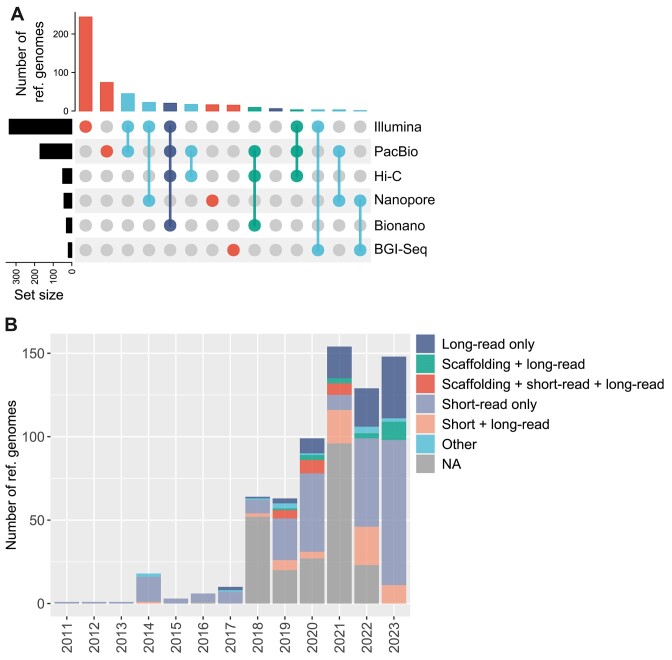
Available reference genomes by sequencing technology and year of release. (A) An upset plot showing the frequency of all reference genomes for marine vertebrates to date according to submitter-reported sequencing technologies used for their generation. Colors indicate 1 (red), 2 (cyan), 3 (green), or 4 (navy) technologies used, respectively. Data are missing for 219 assemblies (31%); these assemblies are not shown. (B) The frequencies of reference genome assembly releases according to both year of release and combinations of technology types; scaffolding refers to Hi-C and/or Bionano, short-read refers to Illumina and/or BGI-Seq, and long-read refers to PacBio and/or Nanopore technology.

**Figure 2:**
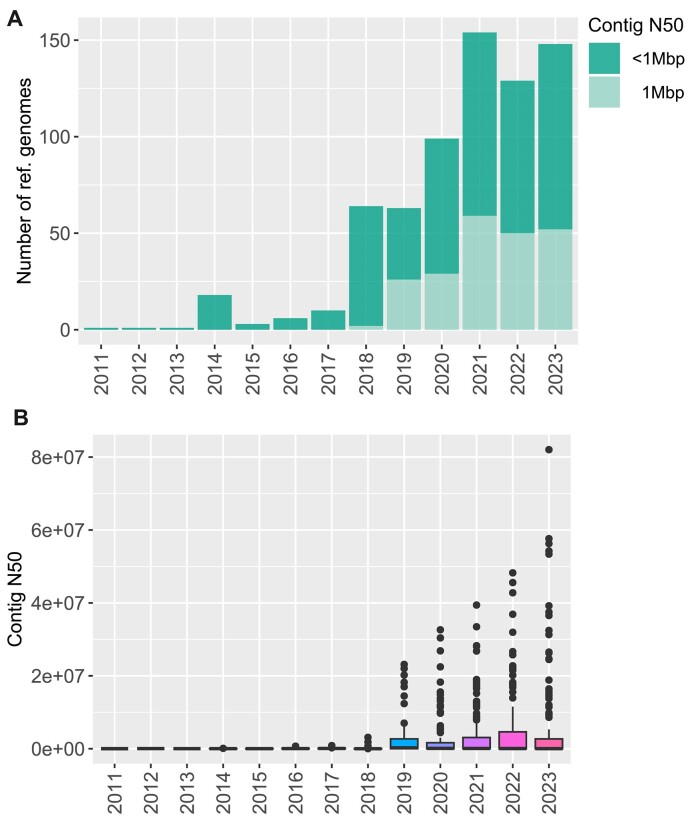
The contiguity of available reference genomes by year of release. The frequency of assemblies for marine vertebrates with a contig N50 >1 Mbp by year of release (A) and box plots of contig N50 values by year of release (B).

We next sought to characterize available reference genomes in terms of their quality and the sequencing technologies used for their generation. Noting that data on technology type are submitter-defined and were unavailable for 219 assemblies (31%), Illumina short-read sequencing was most common (*n* = 337), followed by Pacific Biosciences long-read sequencing (*n* = 171, Fig. 1A). This trend remained even when restricting analysis to reference genomes released in 2023 alone (Fig. 1B). Regarding contiguity, the production of high-contiguity genomes (contig N50 >1 Mbp) is accelerating (Fig. 2A), along with a general trend of increasing contiguity over time (Fig. 2B). The current dominance of short-read technology likely represents a transitory lag phase, and we expect an imminent shift to long read–based assemblies, scaffolded to chromosome level with Hi-C proximity ligation sequencing, as costs decline and accessibility improves [7].

## Data Production Is Biased toward Higher-Resourced Regions

**Figure 3:**
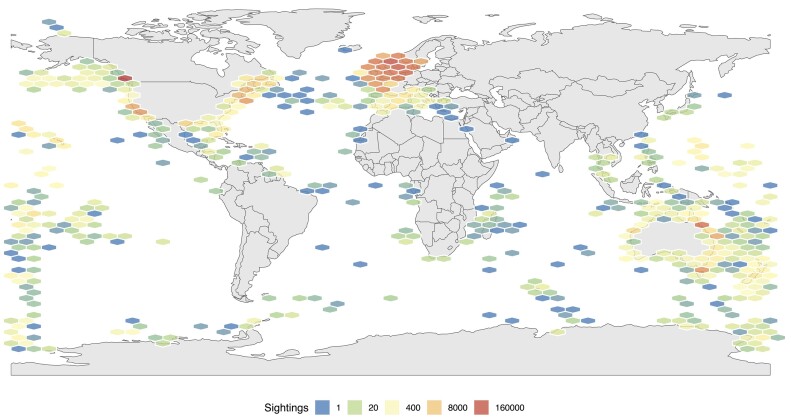
Global sightings of marine vertebrates with reference genomes. The list of species for which a reference genome is available on NCBI was cross-referenced with all sightings for these species collated by the Ocean Biodiversity Information System (OBIS). The total number of sightings is represented here. For ease of visualization, the OBIS data were restricted to sightings of ray-finned (Actinopteri) and cartilaginous fish (Elasmobranchii and Holocephali) since 2000.

To examine the geographic distribution of marine vertebrate reference genome resources, we cross-referenced the assembled species with comprehensive sighting data extracted from the Ocean Biodiversity Information System (OBIS) full report [8]. Projecting sightings data onto a world map revealed a clear spatial imbalance favoring fauna occurring in oceans and coastal regions of North America, the United Kingdom, and the east coast of Australia (Fig. 3). Reference genome (and perhaps sightings) data representing the fauna of lower-resourced regions are comparatively lacking. This not only reveals a large data gap but also emphasizes the need for equitable representation across diverse marine regions to ensure a holistic understanding of our global ocean biodiversity.

## Challenges and Opportunities

Remarkable advances in sequencing and computational power are enabling more efficient production of high-quality marine vertebrate reference genomes, but some important challenges to scaling representation remain. The requirement for high-molecular-weight DNA input for long-read sequencing renders many archival samples unsuitable for high-quality reference genome production. Dedicated fresh sampling of marine vertebrates is logistically complex even for common and relatively accessible species. Many threatened and rare species may not be amenable to fresh sampling at all, with opportunities for reference genome assembly limited to species that can be live-sampled or obtained from poorer-quality DNA sources, such as archival collections [9]. This risks biasing genome production toward common species that are accessible to well-resourced data producers, exacerbating the gaps in our understanding of marine biodiversity and constraining the conservation management utility of reference genome–enabled research. Harmonization of global initiatives is required to reduce duplication of effort, maximize resource efficiency, and ensure equitable representation of fauna across the phylogeny and diverse marine regions. Toward this goal, Genomes on a Tree [10] aims to synthesize metadata across all genome projects, which importantly includes assembly progress and data for Earth BioGenome Project (EBP) species and affiliated large-scale projects [1]. Enabling dedicated hubs for local data production for underrepresented groups or geographic regions will also facilitate better representation of global marine biodiversity, including rare or threatened taxa with restricted distributions. Adhering to best practices of generating such resources in the place of sample provenance is important to promote fair and equitable sharing of benefits arising from the use of genetic resources [2, 5]. For example, the new EBP-affiliated project *Ocean Genomes* (https://www.minderoo.org/oceanomics) has a specific focus on Southern Hemisphere marine vertebrates, particularly Indian and Indo-West Pacific fauna.

## Conclusions

The convergence of extended read lengths and high accuracy base calling represents a paradigm shift in sequencing technology that has enabled a dramatic improvement in both the rate and quality of reference genome creation. Nevertheless, a considerable data gap remains, with over 96% of marine vertebrate species currently lacking a reference genome. By harnessing advancements in technology and bioinformatics, resource building in underrepresented regions, and continued global coordination and standardization of efforts, the UN Decade of Ocean Science for Sustainable Development can also be the decade of marine vertebrate genomes.

## Abbreviations

EBP: Earth BioGenome Project; NCBI: National Center for Biotechnology Information; OBIS:, Ocean Biodiversity Information System; WoRMS: World Register of Marine Species.

## Supplementary Material

giad119_GIGA-D-23-00279_Original_Submission

giad119_GIGA-D-23-00279_Revision_1

giad119_Response_to_Reviewer_Comments_Original_Submission

giad119_Reviewer_1_Report_Original_Submission Qiong Shi, PhD -- 9/27/2023 Reviewed

giad119_Reviewer_2_Report_Original_Submission Merly Escalona, PhD -- 10/31/2023 Reviewed

giad119_Supplemental_Table

## Data Availability

All code and data required to reproduce these findings are available at GitHub (https://github.com/e-dejong/GigaScience-marine-genomes).

## Additional Files

**Supplementary Table S1**. Available NCBI-listed reference genomes for marine vertebrates.

## Authors’ Information

E.J., L.P., P.B., S.C., and R.E. are part of *Ocean Genomes* (https://www.minderoo.org/oceanomics), a partnership between Minderoo Foundation and the University of Western Australia. *Ocean Genomes* is affiliated with the Earth BioGenome Project and aligning with the protocols, standards, and quality metrics of the Vertebrate Genome Project to accelerate and scale the production of openly accessible reference genome assemblies for marine vertebrates (NCBI BioProject: PRJNA1046164). Based in Perth, Australia, *Ocean Genomes* has a focus on the Southern Hemisphere, particularly Indian and Indo-West Pacific fauna. Threatened and species with high conservation value will also be prioritized.
