# Supplementary material for: Toward genome assemblies for all marine vertebrates: current landscape and challenges
Source: Gigascience. 2024 Jan 27;13:giad119. doi: 10.1093/gigascience/giad119 (PMC10821707; doi:10.1093/gigascience/giad119)
Supplement: giad119_GIGA-D-23-00279_Original_Submission [file giad119_giga-d-23-00279_original_submission.pdf]

GigaScience

Toward genome assemblies for all marine vertebrates: current landscape and challenges

--Manuscript Draft--

**Manuscript Number:**	GIGA-D-23-00279
**Full Title:**	Toward genome assemblies for all marine vertebrates: current landscape and challenges
**Article Type:**	Commentary
**Funding Information:**	
**Abstract:**	Marine vertebrate biodiversity is fundamental to ocean ecosystem health, but is threatened by climate change, overharvesting and habitat degradation. High-quality reference genomes are valuable foundational scientific resources that can inform conservation efforts. Consequently, global consortia are striving to produce reference genomes for representatives of all life. Here, we summarise the current landscape of available marine vertebrate reference genomes including their phylogenetic diversity and geographic hotspots of production. We discuss key logistical and technical challenges that remain to be overcome if we are to realise the vision of a comprehensive reference genome library of all marine vertebrates.
**Corresponding Author:**	Emma de Jong<br>University of Western Australia<br>Crawley, AUSTRALIA
**Corresponding Author Secondary Information:**	
**Corresponding Author's Institution:**	University of Western Australia
**Corresponding Author's Secondary Institution:**	
**First Author:**	Emma de Jong
**First Author Secondary Information:**	
**Order of Authors:**	Emma de Jong
	Lara Parata
	Philipp E Bayer
	Shannon Corrigan
	Richard J Edwards
**Order of Authors Secondary Information:**	
**Additional Information:**	
**Question**	**Response**
Are you submitting this manuscript to a special series or article collection?	No
**Experimental design and statistics**<br><br>Full details of the experimental design and statistical methods used should be given in the Methods section, as detailed in our Minimum Standards Reporting Checklist. Information essential to interpreting the	No

Powered by Editorial Manager® and ProduXion Manager® from Aries Systems Corporation

data presented should be made available in the figure legends.<br><br>Have you included all the information requested in your manuscript?	
If not, please give reasons for any omissions below.<br><br>as follow-up to "**Experimental design and statistics**<br><br>Full details of the experimental design and statistical methods used should be given in the Methods section, as detailed in our Minimum Standards Reporting Checklist. Information essential to interpreting the data presented should be made available in the figure legends.<br><br>Have you included all the information requested in your manuscript?<br><br>"	Full details of the methods are described throughout the main text of the commentary, not in a dedicated methods section.
**Resources**<br><br>A description of all resources used, including antibodies, cell lines, animals and software tools, with enough information to allow them to be uniquely identified, should be included in the Methods section. Authors are strongly encouraged to cite Research Resource Identifiers (RRIDs) for antibodies, model organisms and tools, where possible.<br><br>Have you included the information requested as detailed in our Minimum Standards Reporting Checklist?	No
If not, please give reasons for any omissions below.<br><br>as follow-up to "**Resources**	Full details of the methods are described throughout the main text of the commentary, not in a dedicated methods section.

A description of all resources used, including antibodies, cell lines, animals and software tools, with enough information to allow them to be uniquely identified, should be included in the Methods section. Authors are strongly encouraged to cite Research Resource Identifiers (RRIDs) for antibodies, model organisms and tools, where possible.<br><br>Have you included the information requested as detailed in our Minimum Standards Reporting Checklist?<br><br>"	
**Availability of data and materials**<br><br>All datasets and code on which the conclusions of the paper rely must be either included in your submission or deposited in publicly available repositories (where available and ethically appropriate), referencing such data using a unique identifier in the references and in the “Availability of Data and Materials” section of your manuscript.<br><br>Have you have met the above requirement as detailed in our Minimum Standards Reporting Checklist?	Yes

Powered by Editorial Manager® and ProduXion Manager® from Aries Systems Corporation

Manuscript

Click here to access/download;Manuscript;GigaScience_deJong_submitted.

***Title**: Toward genome assemblies for all marine vertebrates: current landscape and challenges*

Emma de Jong[1], Lara Parata[1], Philipp E. Bayer[1,2], Shannon Corrigan[1,2], Richard J. Edwards[1,3]

[1]Minderoo OceanOmics Centre at UWA, Oceans Institute, University of Western Australia, Perth, Australia
[2]Minderoo Foundation, Perth, Australia
[3]Evolution and Ecology Research Centre, School of Biotechnology and Biomolecular Sciences, University of New South Wales, Sydney, Australia

Corresponding author: Emma de Jong: emma.dejong@uwa.edu.au
Email addresses for co-authors: lara.parata@uwa.edu.au, pbayer@minderoo.org, scorrigan@minderoo.org, rich.edwards@uwa.edu.au

ORCiDs: EDJ = 0000-0002-2501-6119; LP = 0000-0003-2408-6788; PEB = 0000-0001-8530-3067; SC = 0000-0003-0093-5028; RJE = 0000-0002-3645-5539

Keywords: genomics, vertebrates, marine, reference genome, Actinopterygii, Chondrichthyes, biodiversity

Word count (including abstract): 1136

Abbreviations

Earth BioGenome Project, EBP
NCBI, National Center for Biotechnology Information
OBIS, Ocean Biodiversity Information System
WoRMS, World Register of Marine Species

Abstract (96 words)

Marine vertebrate biodiversity is fundamental to ocean ecosystem health, but is threatened by climate change, overharvesting and habitat degradation. High-quality reference genomes are valuable foundational scientific resources that can inform conservation efforts. Consequently, global consortia are striving to produce reference genomes for representatives of all life. Here, we summarise the current landscape of available marine vertebrate reference genomes including their phylogenetic diversity and geographic hotspots of production. We discuss key logistical and technical challenges that remain to be overcome if we are to realise the vision of a comprehensive reference genome library of all marine vertebrates.

Background (165 words)

Reference genomes have become a fundamental tool for modern biology: reference genome-enabled applications have driven discoveries across medicine and healthcare, agriculture, biodiversity, ecology, conservation, and evolution. Reference genomes have been economically, logistically, technically, and computationally challenging to produce, leading to reliance on select model organisms to inform genomics-based research. In recent years, advances in sequencing technology and computational tools have facilitated the rapid and affordable production of reference genomes for non-model organisms across the tree of life, with ambitious global efforts underway to compile reference genomes for all eukaryotes [1]. The enhanced capacity for large-scale production of reference genomes is timely, as inferences from reference genome-enabled research can inform conservation management practice in this period of unprecedented biodiversity loss and ecosystem decline [2]. Here, we discuss the current landscape of available reference genomes with a focus on marine vertebrates in light of global recognition of the critical role that the oceans and marine biodiversity play in stabilising our climate and supporting a blue economy [3].

Main text (875 words)

Reference genomes are unavailable for over 96% of marine vertebrate species

We assessed the number and phylogenetic diversity of reference genomes currently available for marine vertebrate species. Metadata for assemblies categorised as reference-level were obtained from the National Center for Biotechnology Information (NCBI) via their Datasets command line tool using “chordates” as the query. Resulting entries were cross-referenced with all known marine vertebrate species (*n*=19,800) from the World Register of Marine Species [4], yielding a final dataset of 697 assemblies representing 688 unique species (Supplementary Table 1). Eighty-four percent of marine vertebrate orders are represented by at least one species (78/93, Table 1), highlighting the progress of existing global consortia with early strategies to target order-level representatives [5]. Representation rapidly diminishes at lower taxonomic levels, however, covering only 41% of marine vertebrate families, 12% of genera, and 3.5% of species. Perciformes, the most speciose vertebrate order, has the highest number of reference genomes, yet still only 39% of Perciformes families are represented. Furthermore, orders with the highest percentage of threatened species according to the IUCN Red List are amongst the least represented. For example, 47 Rhinopristiformes species are listed as threatened [6], yet currently only 2 species from this order are represented by a reference genome. These data emphasise the need for continued efforts to capture the rich diversity of marine vertebrates, particularly the most vulnerable taxa that are likely to be of high conservation value.

Available genomes are predominantly derived from short-read sequencing technology

We next sought to characterise available reference genomes in terms of their quality and the sequencing technologies used for their generation. Noting that data on technology type is submitter-defined, and was unavailable for 219 assemblies (31%), Illumina short-read sequencing was most common (*n*=337), followed by Pacific Biosciences long-read sequencing (*n*=171, Figure 1A). This trend remained even when restricting analysis to reference genomes released in 2023 alone (Figure 1B). Regarding contiguity, the production of high-contiguity genomes (contig N50 >1Mbp) is accelerating (Figure 2A), along with a general trend of increasing contiguity over time (Figure 2B). The current dominance of short-read technology likely represents a transitory lag phase, and we expect an imminent shift to long-read-based assemblies as costs decline and accessibility improves [7].

Data production is biased toward higher-resourced regions

To examine the geographic distribution of marine vertebrate reference genome resources, we cross-referenced the assembled species with comprehensive sighting data extracted from the Ocean Biodiversity Information System (OBIS) full report [8]. Projecting sightings data onto a world map revealed a clear spatial imbalance favouring fauna occurring in oceans and coastal regions of North America, the United Kingdom, and the east coast of Australia (Figure 3). Reference genome (and perhaps sightings) data representing the fauna of lower-resourced regions is comparatively lacking. This not only reveals a large data gap but emphasises the need for equitable representation across diverse marine regions to ensure a holistic understanding of our global ocean biodiversity.

Challenges and opportunities

Remarkable advances in sequencing and computational power are enabling more efficient production of high-quality marine vertebrate reference genomes, but some important challenges to scaling representation remain. The requirement for high-molecular-weight DNA input for long-read sequencing renders many archival samples unsuitable for high-quality reference genome production. Dedicated fresh sampling of marine vertebrates is logistically complex even for common and relatively accessible species. Many threatened and rare species may not be amenable to fresh sampling at all, with opportunities for reference genome assembly limited to species which can be live-sampled or obtained from poorer quality DNA sources, such as archival collections [9]. This risks biasing genome production toward common species that are accessible to well-resourced data producers, exacerbating the gaps in our understanding of marine biodiversity and constraining the conservation management utility of reference genome-enabled research. Harmonisation of global initiatives is required to reduce duplication of effort, maximise resource efficiency, and ensure equitable representation of fauna across the phylogeny and diverse marine regions. Towards this goal, Genomes on a Tree [10] aims to synthesise metadata across all genome projects, and importantly, includes data on Earth BioGenome Project (EBP) species [1] with genome assemblies currently in progress. Enabling dedicated hubs for local data production for underrepresented groups or geographic regions will also facilitate better representation of global marine biodiversity, including rare or threatened taxa with restricted distributions. Adhering to best practices of generating such resources in the place of sample provenance is important to promote fair and equitable sharing of benefits arising from the use of genetic resources [2, 5]. For example, the EBP-affiliated project *Ocean Genomes*, a partnership between Minderoo Foundation and The University of Western Australia based in Perth, aims to scale

reference genome production for marine vertebrates with a focus on southern hemisphere, particularly Indian and Indo-West Pacific fauna.

Conclusions

The convergence of extended read lengths and high accuracy base calling represents a paradigm-shift in sequencing technology that has enabled a dramatic improvement in both the rate and quality of reference genome creation. Nevertheless, a considerable data gap remains, with over 96% of marine vertebrate species currently lacking a reference genome. By harnessing advancements in technology and bioinformatics, resource building in underrepresented regions, and continued global coordination and standardisation of efforts, the UN Decade of Ocean Science for Sustainable Development can also be the decade of marine vertebrate genomes.

Declarations

Ethics approval and consent to participate

Not applicable.

Consent for publication

Not applicable.

Data Availability

All code and data required to reproduce these findings are available at: https://github.com/e-dejong/GigaScience-marine-genomes

Competing interests

The authors declare that they have no competing interests.

Funding

Not applicable.

Authors' contributions

EJ obtained and analysed the NCBI-listed assembly metadata, compiled the list of marine species from WoRMS, and drafted the manuscript. LP contributed to drafting and critical review of the manuscript and structured the content for the summary table. PB performed analysis on the OBIS data, contributed to interpretation of all results and critically revised the manuscript. SC contributed to interpretation of all results, drafting, and critical revision of the manuscript. RE obtained the raw data from WoRMS, and contributed to interpretation of all results, drafting, and critical revision of the manuscript. All authors read and approved the final manuscript.

Acknowledgements

Not applicable.

Authors' information (optional)

References

1. Lewin, H.A., et al., *Earth BioGenome Project: Sequencing life for the future of life.* Proceedings of the National Academy of Sciences, 2018. **115**(17): p. 4325-4333.
2. Formenti, G., et al., *The era of reference genomes in conservation genomics.* Trends in Ecology & Evolution, 2022. **37**(3): p. 197-202.
3. Ryabinin, V., et al., *The UN Decade of Ocean Science for Sustainable Development.* Frontiers in Marine Science, 2019. **6**.
4. Ahyong, S., et al., *World Register of Marine Species (WoRMS).* 2023.
5. Rhie, A., et al., *Towards complete and error-free genome assemblies of all vertebrate species.* Nature, 2021. **592**(7856): p. 737-+.
6. IUCN. *The IUCN Red List of Threatened Species. Version 2022-2.* 2022 05/09/2023]; Available from: https://www.iucnredlist.org.
7. Li, H. and R. Durbin, *Genome assembly in the telomere-to-telomere era.* arXiv preprint arXiv:2308.07877, 2023.
8. OBIS. *Ocean Biodiversity Information System. Intergovernmental Oceanographic Commission of UNESCO*. 2023; Available from: www.obis.org.
9. Raxworthy, C.J. and B.T. Smith, *Mining museums for historical DNA: advances and challenges in museomics.* Trends in Ecology & Evolution, 2021. **36**(11): p. 1049-1060.
10. Challis, R., et al., *Genomes on a Tree (GoaT): A versatile, scalable search engine for genomic and sequencing project metadata across the eukaryotic tree of life.* Wellcome Open Res, 2023. **8**: p. 24.

Table 1. Summary of the NCBI-listed reference genomes available for marine vertebrates by Order

Class	Order	Marine species	Species with reference genome	Percentage with reference genome	*IUCN Red List % Threatened*[1] *[6]*
Myxini	Myxiniformes	89	1	1.1	11.8
Petromyzonti	Petromyzontiformes	9	4	44.4	21.1
Elasmobranchii	Carcharhiniformes	300	6	2	32.9
Elasmobranchii	Echinorhiniformes	2	0	0	NA
Elasmobranchii	Heterodontiformes	9	0	0	0.0
Elasmobranchii	Hexanchiformes	6	0	0	14.3
Elasmobranchii	Lamniformes	16	2	12.5	66.7
Elasmobranchii	Myliobatiformes	213	2	0.9	48.6
Elasmobranchii	Orectolobiformes	46	6	13	37.8
Elasmobranchii	Pristiophoriformes	10	0	0	0.0
Elasmobranchii	Rajiformes	317	2	0.6	15.5
Elasmobranchii	Rhinopristiformes	87	2	2.3	72.3
Elasmobranchii	Squaliformes	145	2	1.4	22.1
Elasmobranchii	Squatiniformes	26	0	0	59.1
Elasmobranchii	Torpediniformes	70	0	0	42.9
Holocephali	Chimaeriformes	58	2	3.4	7.6
Actinopteri	Acanthuriformes	444	22	5	NA
Actinopteri	Acipenseriformes	16	2	12.5	92.6
Actinopteri	Acropomatiformes	284	1	0.4	NA
Actinopteri	Albuliformes	11	2	18.2	10.0
Actinopteri	Alepocephaliformes	142	0	0	NA
Actinopteri	Anabantiformes	2	0	0	NA
Actinopteri	Anguilliformes	1010	10	1	0.9
Actinopteri	Argentiniformes	97	1	1	NA
Actinopteri	Ateleopodiformes	14	1	7.1	0.0
Actinopteri	Atheriniformes	110	5	4.5	43.6
Actinopteri	Aulopiformes	296	1	0.3	0.0
Actinopteri	Batrachoidiformes	78	2	2.6	18.4
Actinopteri	Beloniformes	184	3	1.6	13.1
Actinopteri	Beryciformes	125	4	3.2	1.4
Actinopteri	Blenniiformes	951	2	0.2	NA
Actinopteri	Callionymiformes	214	2	0.9	NA
Actinopteri	Carangaria incertae sedis	85	3	3.5	NA
Actinopteri	Carangiformes	176	14	8	NA
Actinopteri	Centrarchiformes	171	2	1.2	NA
Actinopteri	Cichliformes	5	1	20	NA
Actinopteri	Clupeiformes	317	11	3.5	6.9
Actinopteri	Cypriniformes	5	0	0	24.3
Actinopteri	Cyprinodontiformes	24	3	12.5	40.3
Actinopteri	Dactylopteriformes	14	1	7.1	NA
Actinopteri	Elopiformes	9	2	22.2	11.1

Class	Order	Marine species	Species with reference genome	Percentage with reference genome	*IUCN Red List % Threatened*[1] *[6]*
Actinopteri	Eupercaria incertae sedis	1784	28	1.6	NA
Actinopteri	Gadiformes	645	45	7	2.6
Actinopteri	Galaxiiformes	8	0	0	NA
Actinopteri	Gobiesociformes	177	2	1.1	11.7
Actinopteri	Gobiiformes	1614	13	0.8	11.0
Actinopteri	Gonorynchiformes	6	1	16.7	14.7
Actinopteri	Holocentriformes	93	4	4.3	NA
Actinopteri	Kurtiformes	372	11	3	NA
Actinopteri	Lampriformes	27	4	14.8	0.0
Actinopteri	Lophiiformes	406	3	0.7	2.0
Actinopteri	Mugiliformes	72	5	6.9	1.9
Actinopteri	Mulliformes	100	1	1	NA
Actinopteri	Myctophiformes	267	2	0.7	0.0
Actinopteri	Notacanthiformes	28	1	3.6	0.0
Actinopteri	Ophidiiformes	562	3	0.5	2.2
Actinopteri	Osmeriformes	33	6	18.2	30.4
Actinopteri	Ovalentaria incertae sedis	798	10	1.3	NA
Actinopteri	Perciformes	3243	168	5.2	9.8
Actinopteri	Pleuronectiformes	792	19	2.4	1.8
Actinopteri	Polymixiiformes	11	1	9.1	0.0
Actinopteri	Saccopharyngiformes	28	0	0	0.0
Actinopteri	Salmoniformes	52	10	19.2	47.4
Actinopteri	Scombriformes	266	9	3.4	NA
Actinopteri	Scorpaeniformes	13	0	0	3.2
Actinopteri	Siluriformes	121	37	30.6	14.4
Actinopteri	Stomiiformes	451	1	0.2	0.0
Actinopteri	Stylephoriformes	1	1	100	NA
Actinopteri	Syngnathiformes	311	26	8.4	6.0
Actinopteri	Tetraodontiformes	415	9	2.2	4.3
Actinopteri	Trachichthyiformes	68	6	8.8	NA
Actinopteri	Zeiformes	34	2	5.9	0.0
Coelacanthi	Coelacanthiformes	2	1	50	100.0
Aves	Accipitriformes	1	1	100	22.6
Aves	Anseriformes	48	7	14.6	14.7
Aves	Charadriiformes	273	30	11	13.2
Aves	Ciconiiformes	12	1	8.3	25.0
Aves	Coraciiformes	3	1	33.3	9.1
Aves	Falconiformes	5	3	60	12.1
Aves	Gaviiformes	5	1	20	0.0
Aves	Gruiformes	1	0	0	25.4
Aves	Pelecaniformes	61	10	16.4	16.4
Aves	Podicipediformes	15	2	13.3	21.7
Aves	Procellariiformes	138	10	7.2	44.9

Class	Order	Marine species	Species with reference genome	Percentage with reference genome	*IUCN Red List % Threatened*[1] *[6]*
Aves	Sphenisciformes	20	18	90	50.0
Crocodylia	NA	2	1	50	NA
NA	Sauria	1	0	0	NA
NA	Squamata	84	6	7.1	16.7
NA	Testudines	7	4	57.1	63.0
Mammalia	Carnivora	44	14	31.8	26.3
Mammalia	Cetartiodactyla	90	33	36.7	36.3
Mammalia	Sirenia	4	2	50	80.0

1. IUCN threatened species includes critically endangered, endangered, and vulnerable species. Red text highlights those orders where % threatened species > % with an available reference genome.

Figure legends

Figure 1. Available reference genomes by sequencing technology and year of release. A) An upset plot showing the frequency of all reference genomes for marine vertebrates to date according to submitter-reported sequencing technologies used for their generation. Colours indicate one (red), two (cyan), three (green) or four (navy) technologies used, respectively. Data is missing for 219 assemblies (31%), these assemblies are not shown. B) The frequencies of reference genomes assembly releases according to both year of release and combinations of technology types; scaffolding refers to Hi-C and/or Bionano, short-read refers to Illumina and/or BGI-Seq, and long-read refers to PacBio and/or Nanopore technology.

Figure 2. The contiguity of available reference genomes by year of release. The frequency of assemblies for marine vertebrates with a contig N50 > 1Mbp by year of release (A), and box plots of contig N50 values by year of release (B).

Figure 3. Global sightings of marine vertebrates with reference genomes. The list of species for which a reference genome is available on NCBI was cross-referenced with all sightings for these species collated by Ocean Biodiversity Information System (OBIS). The total number of sightings is represented here. For ease of visualisation, the OBIS data was restricted to sightings of ray-finned (Actinopteri), and cartilaginous fish (Elasmobranchii and Holocephali) since the year 2000.

Figure 1

Click here to access/download;Figure;Fig1-final.png

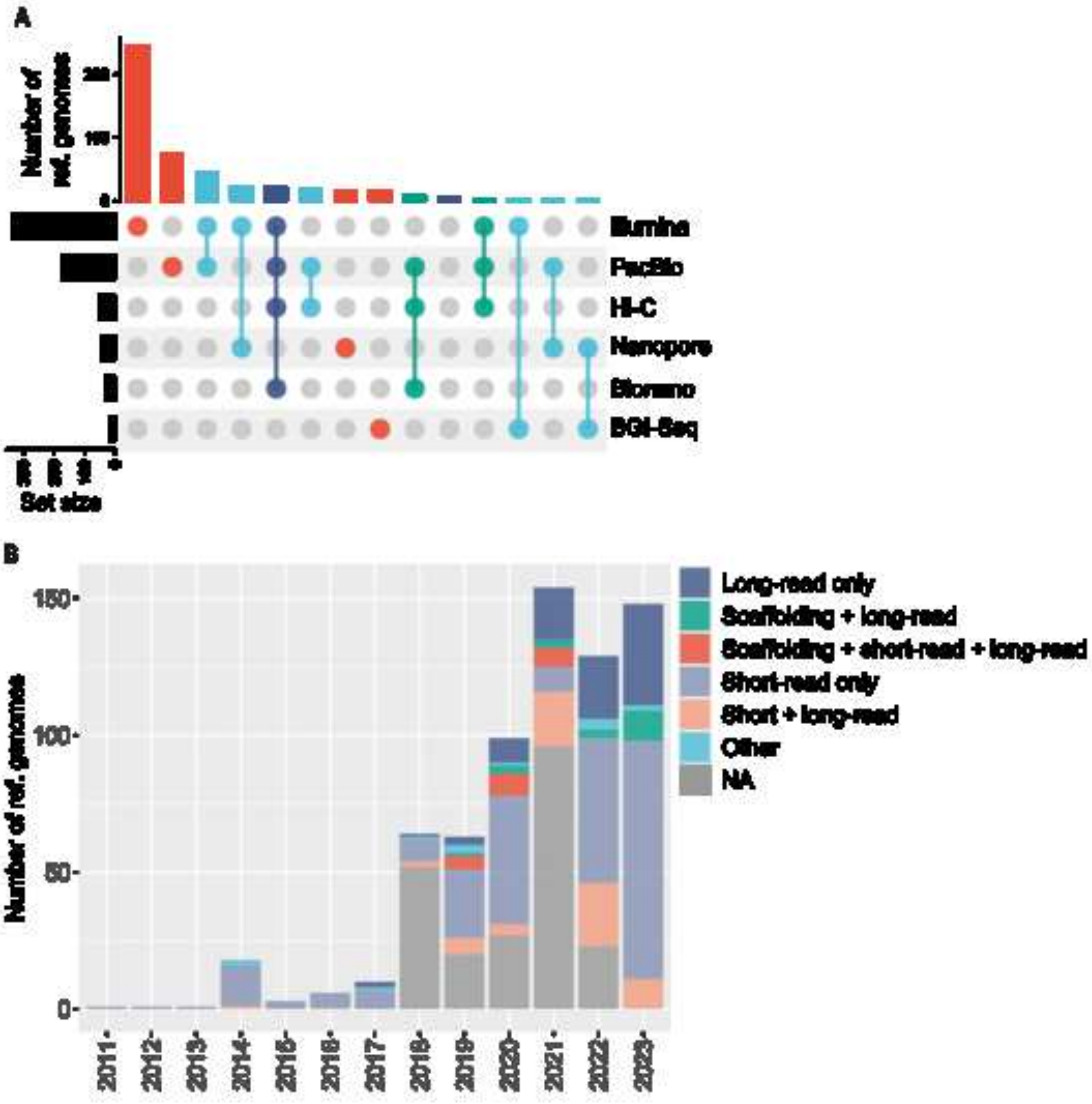

Figure 2

Click here to access/download;Figure;Fig2-final.png

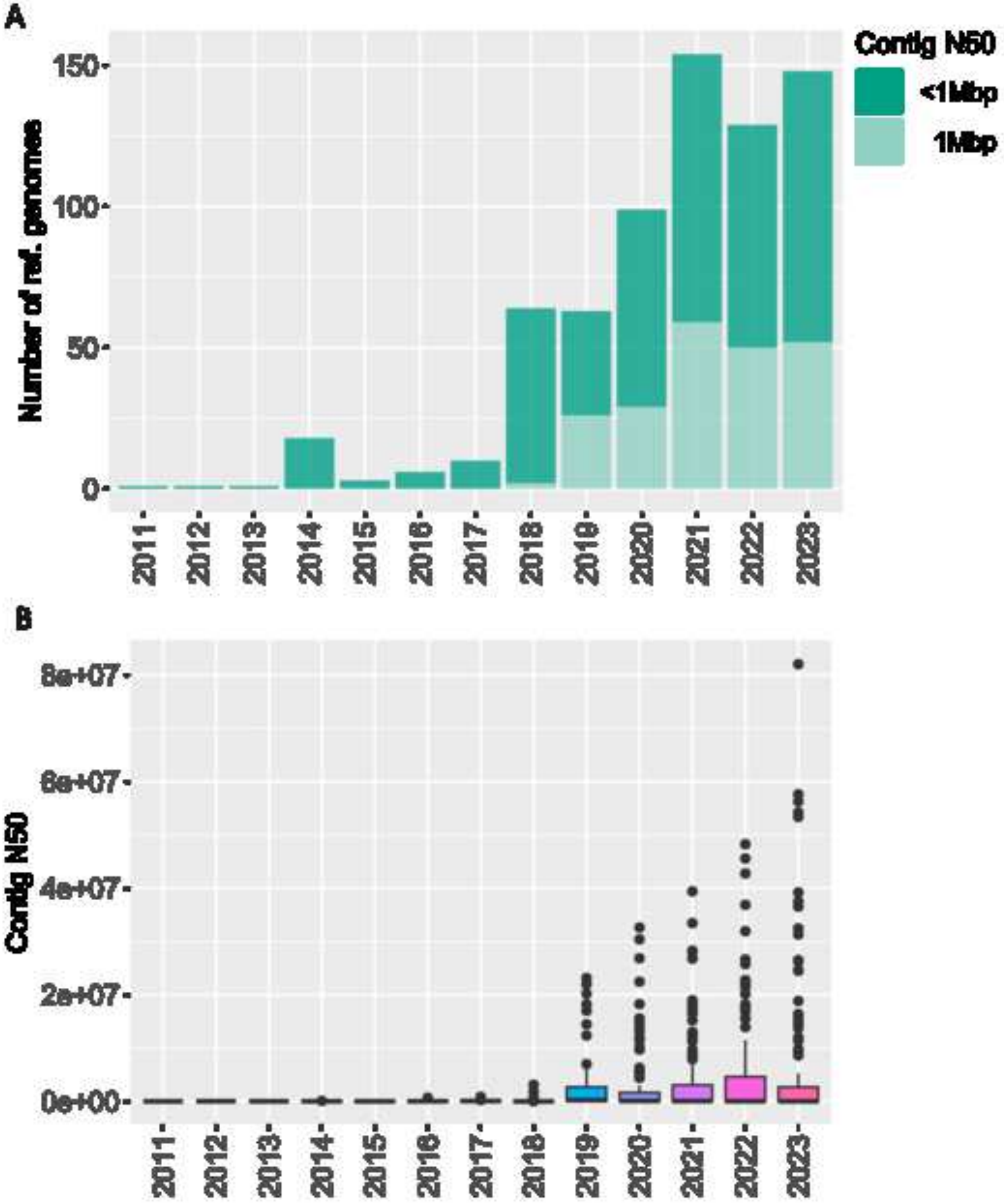

Figure 3

Click here to access/download;Figure;Fig3.png

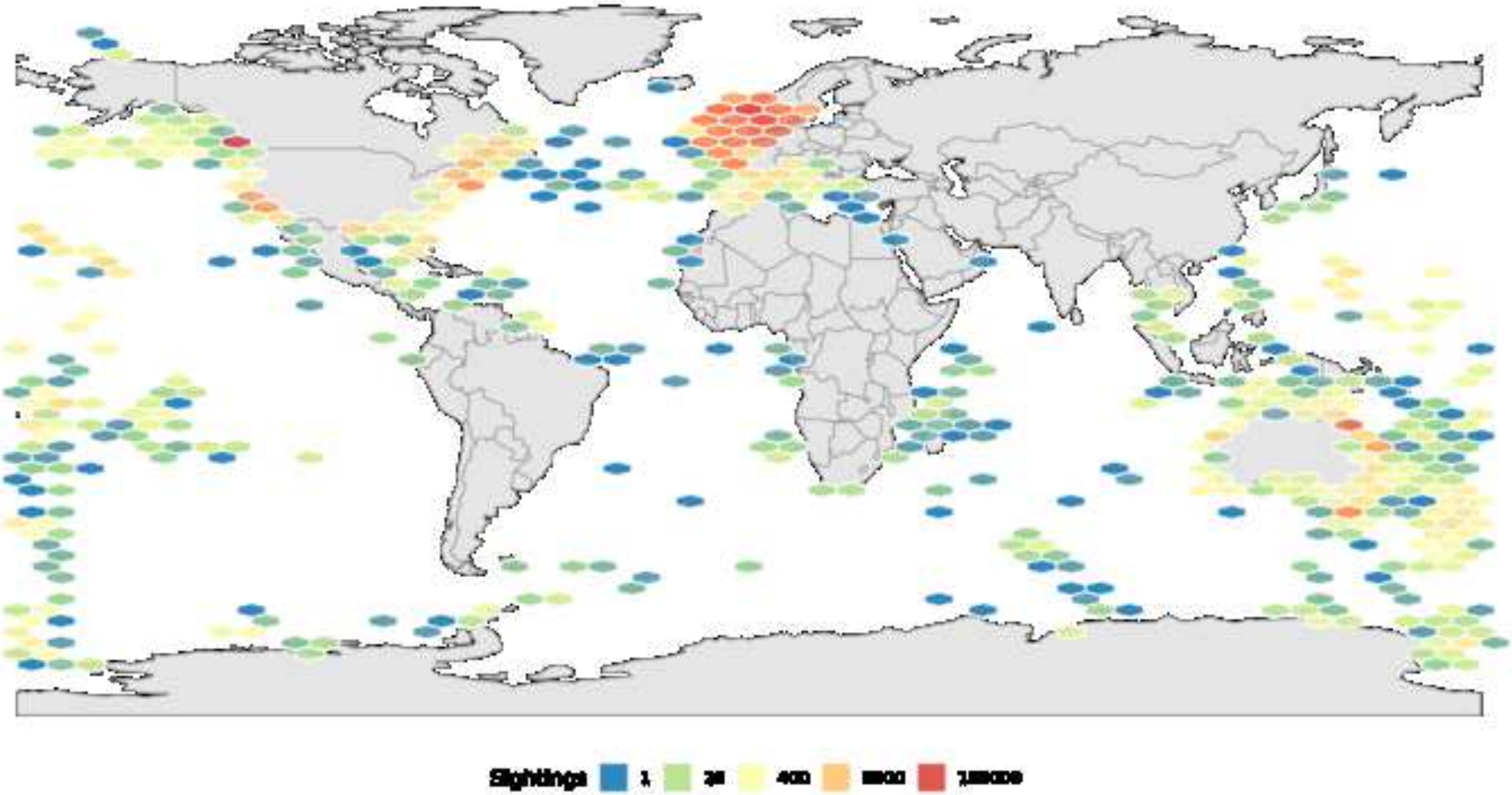

Supplementary Table 1

Click here to access/download
Supplementary Material
SupplementaryTable1.xlsx
