# Supplementary material for: Toward genome assemblies for all marine vertebrates: current landscape and challenges
Source: Gigascience. 2024 Jan 27;13:giad119. doi: 10.1093/gigascience/giad119 (PMC10821707; doi:10.1093/gigascience/giad119)
Supplement: giad119_Response_to_Reviewer_Comments_Original_Submission [file giad119_response_to_reviewer_comments_original_submission.pdf]

Author's Response To Reviewer Comments

Dear Editor,

RE: Response to Reviewer Comments - GIGA-D-23-00279

Thank you for the opportunity to submit an updated revision to our manuscript, " Toward genome assemblies for all marine vertebrates: current landscape and challenges " (GIGA-D-23-00279). We have made the requested revisions, listed in detail below.

Yours sincerely,
Dr. Emma de Jong
Postdoctoral Research Fellow, Ocean Genomes Laboratory
Minderoo OceanOmics Centre at UWA
UWA Oceans Institute

Reviewer #1

Comment 1: As a brief commentary, this manuscript talks mainly about current landscape and challenges for marine vertebrates' genome assemblies. In general, the overall writing is good, and numerous data were collected to support the main conclusions. Only minor revisions are required before acceptance for publication. Extra editing is necessary. For example, under the section of "Challenges and opportunities", provide the full name for EBP.

Response 1: We have checked the text to ensure all abbreviations are defined. The full name for EBP was provided on the title page, as well as at first use in the text: "…, includes data on Earth BioGenome Project (EBP) species [1]…"

Comment 2: Under the section of "Available genomes are predominantly derived from short-read sequencing technology": The authors are recommended to mention the current status of increasing availability of chromosome-level genome assemblies based on the Hi-C sequencing technology, although Hi-C appears in the Fig. 1.

Response 2: We agree with the reviewer that this is of interest but felt uncomfortable with the criteria for calling assemblies "chromosome-level" within the space constraints of this commentary. It is something we intend to expand upon in a future review. We have increased the emphasis by expanding the last sentence: "The current dominance of short-read technology likely represents a transitory lag phase, and we expect an imminent shift to long-read-based assemblies, scaffolded to chromosome-level with Hi-C proximity ligation sequencing, as costs decline and accessibility improves [7]."

Comment 3: Therefore, under the section of "Challenges and opportunities", the authors may provide short discussions about the difficult in Hi-C sequencing and comparative genomics analysis (due to available data from different sequencing methods; see Fig. 1) of some marine vertebrates.

Response 3: The requirements for successful Hi-C scaffolding include questions of both sample and assembly quality. Whilst this is clearly of interest, it was beyond the scope and space constraints of this commentary. We have instead concentrated on highlighting more general concerns about available sample quality for sequencing and assembly.

Reviewer #2

The authors underscore the importance of marine vertebrate genome assemblies given the current climate

crisis and considering the role the oceans and marine biodiversity play in its understanding and stabilization and how they would support the blue economy. They highlight the small number of marine vertebrate species available, how the distribution of the species is skewed towards resource rich regions and point out the challenges faced while trying to increase the efforts. I think the manuscript in general follows the guidelines for a Commentary article in this Journal. The manuscript is well written, the methods described are appropriate for the type of study described and the conclusion supports the data shown. However, there are 3 points that I'd like to be addressed.

Comment 1. The manuscript, though following the guidelines of a Commentary article, feels like a presentation of the Ocean Genomes project. Is this the case? 1.1 If so, it would be interesting to have more information about the project on a different type of manuscript. 1.2 If not, it would be important to have a link to the project site (or a link to a site where the details of the project are described). Otherwise, and understanding that there might be a limit with the citations, there are other projects working on marine species that could be cited here as well.

Response 1: Reviewer #2 is correct to suppose that a more detailed description of the Ocean Genomes project will follow. We thought it useful to highlight the project here, and have provide a link as requested, in addition to emphasising that it is new. We have also now shifted some of the details into the Authors' Information section.

"For example, the new EBP-affiliated project Ocean Genomes (https://www.minderoo.org/oceanomics), has a specific focus on southern hemisphere marine vertebrates, particularly Indian and Indo-West Pacific fauna."

We did not add links to more projects due to the citation limit of 10. We have instead tried to emphasise that the EBP umbrella contains many affiliated projects of interest:
"Towards this goal, Genomes on a Tree [10] aims to synthesise metadata across all genome projects, which importantly includes assembly progress and data for Earth BioGenome Project (EBP) species and affiliated large-scale projects [1]."

Comment 2. Given the progress of the different genome projects, and the pace at which the NCBI database is being updated, would it be possible to add when the data has been retrieved?

Response 2: The date has been added.

Comment 3. The resolution of the figures need to be improved. The font size or format makes it hard to read.

Response 3: The figures were supplied at 600 dpi resolution, so this may be a PDF issue. We will supply alternative high-resolution formats if required.

Editor comments: Please add info about missing the BGI T1K fishes project to the revised manuscript.

Response: Whilst we did originally have a section on annotation and transcriptomics, we cut it out due to space constraints. We therefore wondered whether this was supposed to refer to the Fish10K project? Due to the citation limit, we have tried to cover this and other affiliated projects under the blanket coverage of the Earth Biogenome Project (EBP). Please let us know if you would prefer us to increase the citation count.
