# Supplementary material for: Toward genome assemblies for all marine vertebrates: current landscape and challenges
Source: Gigascience. 2024 Jan 27;13:giad119. doi: 10.1093/gigascience/giad119 (PMC10821707; doi:10.1093/gigascience/giad119)
Supplement: giad119_Reviewer_2_Report_Original_Submission — Merly Escalona, PhD -- 10/31/2023 Reviewed [file giad119_reviewer_2_report_original_submission.pdf]

Reviewer Report

Title: Toward genome assemblies for all marine vertebrates: current landscape and challenges

Version: Original Submission **Date:** 10/31/2023

Reviewer name: Merly Escalona, PhD

Reviewer Comments to Author:

Comment to authors.The authors underscore the importance of marine vertebrate genome assemblies given the current climate crisis and considering the role the oceans and marine biodiversity play in its understanding and stabilization and how they would support the blue economy. They highlight the small number of marine vertebrate species available, how the distribution of the species is skewed towards resource rich regions and point out the challenges faced while trying to increase the efforts.I think the manuscript in general follows the guidelines for a Commentary article in this Journal. The manuscript is well written, the methods described are appropriate for the type of study described and the conclusion supports the data shown.However, there are 3 points that I'd like to be addressed.1. The manuscript, though following the guidelines of a Commentary article, feels like a presentation of the Ocean Genomes project. Is this the case?1.1 If so, it would be interesting to have more information about the project on a different type of manuscript.1.2 If not, it would be important to have a link to the project site (or a link to a site where the details of the project are described). Otherwise, and understanding that there might be a limit with the citations, there are other projects working on marine species that could be cited here as well.2. Given the progress of the different genome projects, and the pace at which the NCBI database is being updated, would it be possible to add when the data has been retrieved?3. The resolution of the figures need to be improved. The font size or format makes it hard to read.

Level of Interest

Please indicate how interesting you found the manuscript: Choose an item.

Quality of Written English

Please indicate the quality of language in the manuscript: Choose an item.

Declaration of Competing Interests

Please complete a declaration of competing interests, considering the following questions:

- Have you in the past five years received reimbursements, fees, funding, or salary from an organisation that may in any way gain or lose financially from the publication of this manuscript, either now or in the future?
- Do you hold any stocks or shares in an organisation that may in any way gain or lose financially from the publication of this manuscript, either now or in the future?

- Do you hold or are you currently applying for any patents relating to the content of the manuscript?
- Have you received reimbursements, fees, funding, or salary from an organization that holds or has applied for patents relating to the content of the manuscript?
- Do you have any other financial competing interests?
- Do you have any non-financial competing interests in relation to this paper?

If you can answer no to all of the above, write 'I declare that I have no competing interests' below. If your reply is yes to any, please give details below.

I declare that I have no competing interests

I agree to the open peer review policy of the journal. I understand that my name will be included on my report to the authors and, if the manuscript is accepted for publication, my named report including any attachments I upload will be posted on the website along with the authors' responses. I agree for my report to be made available under an Open Access Creative Commons CC-BY license (http://creativecommons.org/licenses/by/4.0/). I understand that any comments which I do not wish to be included in my named report can be included as confidential comments to the editors, which will not be published.

Choose an item.

To further support our reviewers, we have joined with Publons, where you can gain additional credit to further highlight your hard work (see: https://publons.com/journal/530/gigascience). On publication of this paper, your review will be automatically added to Publons, you can then choose whether or not to claim your Publons credit. I understand this statement.

Yes Choose an item.
